# A Cost-Effectiveness Analysis Evaluating Endoscopic Surveillance for Gastric Cancer for Populations with Low to Intermediate Risk

**DOI:** 10.1371/journal.pone.0083959

**Published:** 2013-12-27

**Authors:** Hui Jun Zhou, Yock Young Dan, Nasheen Naidoo, Shu Chuen Li, Khay Guan Yeoh

**Affiliations:** 1 School of Public Health, Yong Loo Lin School of Medicine, National University of Singapore, Singapore; 2 Department of Medicine, Yong Loo Lin School of Medicine, National University of Singapore, Singapore; 3 Discipline of Pharmacy & Experimental Pharmacology, School of Biomedical Sciences & Pharmacy, University of Newcastle, Callaghan, Australia; Sapporo Medical University, Japan

## Abstract

**Background:**

Gastric cancer (GC) surveillance based on oesophagogastroduodenoscopy (OGD) appears to be a promising strategy for GC prevention. By evaluating the cost-effectiveness of endoscopic surveillance in Singaporean Chinese, this study aimed to inform the implementation of such a program in a population with a low to intermediate GC risk.

**Methods:**

Using a reference strategy of no OGD intervention, we evaluated four strategies: 2-yearly OGD surveillance, annual OGD surveillance, 2-yearly OGD screening and 2-yearly screening plus annual surveillance in Singaporean Chinese aged 50-69 years. From a perspective of the healthcare system, Markov models were built to simulate the life experience of the target population. The models projected discounted lifetime costs ($), quality adjusted life year (QALY), and incremental cost-effectiveness ratio (ICER) indicating the cost-effectiveness of each strategy against a Singapore willingness-to-pay of $46,200/QALY. Deterministic and probabilistic sensitivity analyses were used to identify the influential variables and their associated thresholds, and to quantify the influence of parameter uncertainties respectively.

**Results:**

With an ICER of $44,098/QALY, the annual OGD surveillance was the optimal strategy while the 2-yearly surveillance was the most cost-effective strategy (ICER  =  $25,949/QALY). The screening-based strategies were either extendedly dominated or cost-ineffective. The cost-effectiveness heterogeneity of the four strategies was observed across age-gender subgroups. Eight influential parameters were identified each with their specific thresholds to define the choice of optimal strategy. Accounting for the model uncertainties, the probability that the annual surveillance is the optimal strategy in Singapore was 44.5%.

**Conclusion:**

Endoscopic surveillance is potentially cost-effective in the prevention of GC for populations at low to intermediate risk. Regarding program implementation, a detailed analysis of influential factors and their associated thresholds is necessary. Multiple strategies should be considered in order to recommend the right strategy for the right population.

## Introduction

Mass screening for gastric cancer (GC) has been shown to produce significant improvements in the survival of GC patients [1]–[3]. However, it is still hard to justify the establishment of population-based screening in a country with low to intermediate GC risk because of concerns about cost-effectiveness. Hence, cost-effectiveness evaluations of population-based GC screening are currently limited to jurisdictions with the highest GC incidences in the world, such as Japan, South Korea and Taiwan [4]–[6]. Due to the dramatic impact on cost-effectiveness caused by different levels of GC risk, the findings from these economic evaluations may not be generalizable to other populations.

Endoscopic surveillance, whereby patients with precancerous lesions are closely followed up for GC development by scheduled oesophagogastroduodenoscopy (OGD) examinations, has previously demonstrated the ability to detect GC at an earlier curable stage [7]. Multiple studies have provided evidence of the clinical benefit and cost-effectiveness of endoscopic surveillance in patients with atrophic gastritis, intestinal metaplasia, gastric ulcer or dysplasia [8]–[11]. Thus, the economic feasibility of OGD-based surveillance as a national strategy for GC prevention in countries at low to intermediate risk is worthy of further investigation.

In Singapore, the majority Chinese population is at an intermediate risk of GC [12]. The interest in early detection to improve the survival and quality of life of GC patients has stimulated a series of endeavors. Based on decision-analytic models, Dan et al. previously reported that 2-yearly OGD screening is cost-effective in Singaporean Chinese men aged 50–70 years [13]; while Xie et al. evaluated the primary prevention strategy of *H.pylori* screening and eradication in Singaporean Chinese aged 40 years or older [14]. Additionally, an ongoing hospital-based demonstration project, the Gastric Cancer Epidemiology, Clinical and Genetics Program (GCEP) [15] was initiated in Singapore in 2004 with the intention of providing empirical evidence of the feasibility and cost-effectiveness of endoscopic surveillance.

However, consensus has yet to be reached regarding the optimal strategy for GC prevention in Singapore. Furthermore, none of these aforementioned studies has provided evidence regarding cost-effectiveness as yet. Hence, to address this crucial knowledge gap to assist decision-makers and clinicians, we constructed Markov models to evaluate the cost-effectiveness of OGD-based surveillance and mass screening. Our main objectives were to: (1) inform the choice of optimal strategy for GC prevention within the context of the Singapore heath care system and (2) provide suggestions for actual implementation of an OGD-based surveillance program in a country at low to intermediate GC risk. Our study demonstrated that endoscopic surveillance is cost-effective and is potentially the optimal strategy for GC prevention in a country with low to intermediate GC risk.

## Methods and Materials

### Target Population

The target population was defined as Singaporean Chinese aged 50–69 years based on epidemiologic evidence that this cohort carries 90% of the GC disease burden in Singapore and has a sharp increase in GC risk after the age of 50 years [12].

### Strategies compared

**Figure 1:**
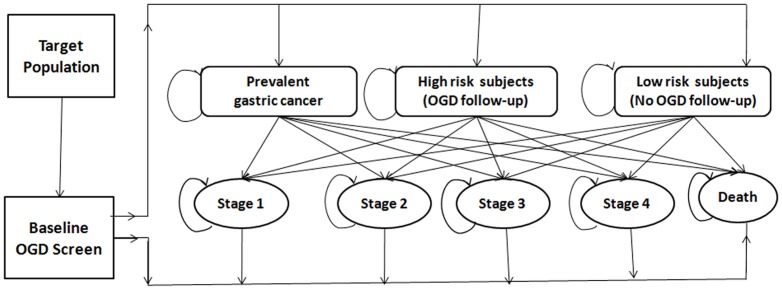
Overview of the surveillance strategy.

An overview of the focused surveillance strategies compared in our study are illustrated in Figure 1. A baseline OGD examination was used to screen the entire target population for high risk subjects, who were defined by the presence of precancerous lesions in the stomach. The high risk group was then subjected to OGD follow-up while subjects without precancerous lesions, the low risk group, remained under usual care. Considering the different progression rates of different premalignancies in the stomach, we evaluated two follow-up frequencies: annual OGD surveillance and 2-yearly OGD surveillance as per the European Society of Gastrointestinal Endoscopy Guidelines for management of precancerous conditions and lesions in the stomach [16].

The screening strategy evaluated in our study was to examine the whole cohort of 50–69 year old Chinese every two years in light of a previous cost-effectiveness analysis by Dan et al [13]. Furthermore we combined 2-yearly screening with annual surveillance as the most intensive strategy to explore the maximum potential of early detection. Finally, using no OGD intervention as the reference strategy, we compared to it the four strategies, namely, 2-yearly OGD surveillance, annual OGD surveillance, 2-yearly OGD screening and 2-yearly OGD screening plus annual OGD surveillance.

### Major Assumptions

To ensure clinical validity of our study, the Markov model was built on the following assumptions.

1) The effect of the four strategies is limited to down-staging due to early detection and thus GC incidence is not affected [1], [17].

2) For the 2-yearly screening and the 2-yearly surveillance strategies that deliver OGD services every other year, the early detection effect persists in the interval years without OGD examination but is less effective. The early detection effect is assumed to be 40% (2-yearly screening) and 60% (2-yearly surveillance) of that conferred by annual OGD follow-up for these interval years.

3) The probability that precancerous lesions could regress to a healthy or less advanced state for high risk subjects is negligible [18].

4) Full subject compliance with the OGD schedule and full adherence to standardized treatment following a positive OGD was assumed.

5) GC patients receive the same standardized treatment after diagnosis and therefore undergo the same survival experience for all five strategies.

### Markov states and utility

Markov states were broadly defined as (a) death (from GC or other causes) with a utility of 0, (b) the four clinical stages, namely GC stage 1, GC stage 2, GC stage 3 and GC stage 4 with the stage-specific utility estimated as EQ-5D scores derived from our previous quality of life study [19], (c) an asymptomatic state assigned with a utility of 1, which encompasses all the remaining Markov states.

### Model Construction and Patient Flow

We adopted a perspective of the health care system for the purpose of making the study informative for program implementation. Individual Markov models were first built for the reference strategy and the four strategies (Figure S1–S5). Each model simulated the life experience of the target population following the clinical pathways specified by the evaluated strategies. A decision tree was used to compare these five Markov models to identify the optimal strategy (Figure S6). Our model discounted both cost and effectiveness at an annual rate of 3% [20].

In each Markov model, the simulation started with the target population being asymptomatic, i.e. the cohort was free of GC but was exposed to GC risk. As the Markov modeling progressed, the cohort developed GC governed by population incidences [21]. In a given Markov model, all the incident GC patients were diagnosed with one of four clinical stages. The distribution of GC stages was determined as per clinical pathway of each strategy. Using different stage proportions in accordance with the predefined OGD proposal, the down-stage effect from screening and surveillance was incorporated into the respective Markov models. The GC cohorts corresponding to the four clinical stages were modeled separately until death. If a subject did not suffer GC in a given cycle, he/she would remain asymptomatic at the start of the next cycle and go through another cycle of the modeling process. The Markov models ran year by year until a minimum of 99% of the target population died.

### Data synthesis

A PubMed literature search was conducted using key terms “endoscopic surveillance”, “precancerous lesions”, “gastric/stomach cancer screening”, “cost-effectiveness analysis” and “economic evaluation”. Each article was assessed in terms of validity, reliability and transferability. As per the generally accepted hierarchy of evidence, systematic reviews and meta-analyses were given the highest priority, followed by randomized control trials, prospective cohort studies and cross-sectional studies. Data from Singapore and other Asia-based studies were used as far as possible. Point estimates and their plausible ranges were presented for each input variable (Table 1).

**Table 1:** Input variables and sources.

Parameters	Base case estimates and Range	Reference
**Epidemiologic data**		
Incidence* (1/100,000)	GC incidences of Singaporean Chinese	[12]
Background mortality* (%)	2011 Life Table of Singaporean Chinese	[21]
Prevalence of premalignancy (%)	13.50 (6.5– 27)	[15], [24]
Odds ratio†	6.0 (2.4–21.5)	[23]
5-Year Survival (Stage 1:2:3:4)	90%:70%:40%:0%	[36]
Stage distribution of GC cohort	(Stage 1:2:3:4)	
Detected by programs	85%:4%:8%:3%	[36], [37]
Detected in usual practice	7%:17%:33%:43%	[25]–[27]
**OGD test characteristics**		
Sensitivity	0.93 (0.44 –0.99)	[28]
Specificity	1 (0.95 – 1)	
**Cost parameters ($)**		
Baseline OGD/Biopsy in surveillance	350 (175–750)	[31]
OGD/Biopsy	340 (170–680)	[21]
Diagnosis & Staging‡	740 (660 – 820)	
Diagnosis & Staging§	1155 (960–1440)	
**Treatment**		
Stage 1	17000 (8500 – 34000)	Hospital Charge 2012
Stage 2	27200 (13600 – 54400)	
Stage 3	38000 (19000 – 76000)	
Stage 4	15500 (7800 – 31100)	
Post-treatment GC follow-up	955 (900–1300)	
Program cost ∥ (%)	40 (20–80)	[31], [32], [38]
**Utilities**		
Stage 1	0.88 (0.60 – 1.00)	[19]
Stage 2	0.86 (0.62–0.99)	
Stage 3	0.77 (0.58 – 0.95)	
Stage 4	0.68 (0.51 – 0.84)	
**Other parameters**		
Discount rate (%)	3 (0–5)	[39]
Willingness to pay ($1000/QALY)	46.2 (15– 100)	[34]

data are age and gender-specific.

Odds ratio for GC of high risk group versus low risk group.

Diagnosis and staging cost for GC cases detected by prevention strategy.

Diagnosis and staging cost for GC cases detected in usual care.

Proportion of total operational cost.

### Epidemiological Data

The background mortality of the target population was represented by the life-tables of the 2011 Singapore population [21]. For the GC patient cohort, the probability of dying from other causes was calculated following the approach used in the Cost of Illness Handbook, United States Environmental Protection Agency [22]. In the Markov trees for the reference strategy and the 2-yearly screening strategy, annual transition rates to GC were represented by population incidences specific to age and gender [12]. In the Markov trees for the two surveillance strategies and the screening plus surveillance strategy, the transition rates were computed based on the population incidences, the odds ratio of GC associated with the high risk cohort [23] and the prevalence of precancerous lesions estimated from the GCEP [15] and a community survey in Singapore [24]. The down-stage effect was projected from studies in Korea and Japan [25]–[27]. The sensitivity and specificity of OGD was integrated into the Markov models for screening and surveillance [28].

### Cost

We estimated the incremental costs incurred in the healthcare sector covering cancer treatment, post-treatment follow-up and operation of a prevention program (Table 1). For cancer treatment, we cost medical services utilization specific to each of the four clinical stages (Table S1) in light of the costing rules in the Cost of Illness Handbook [29]. Hospital charges were obtained from the National University Hospital, the not-for-profit tertiary medical institution where the international algorithm for GC treatment is followed [30]. The post-treatment follow-up costs encompassed all expenditures for diagnostic and therapeutic services after initial acute care. Program operational costs in our model were comprised of two parts, the cost of OGD and biopsy and the program cost for activities such as manpower, case management, quality control, transportation and subjects' salary loss due to program participation [31]. The program cost was represented by its proportion of total operating budget. Practically, this proportion indicates the operating efficiency of an actual program [32], [33]. Costs were expressed as 2012 constant United States dollars ($) at an annual average exchange rate of 1.25 Singapore dollars.

### Statistical Analysis

Following WHO guidelines [34], the Singapore GDP of $46,200 per capita for the year 2011 was determined as the willingness-to-pay (WTP) threshold. A strategy associated with an ICER less than $46,200/QALY is considered cost-effective in Singapore for our study.

Markov models were constructed using TreeAge Pro 2009 (TreeAge Software, Inc., Williamstown M.A., USA). After populating the Markov models, we conducted internal validation against the input GC incidences and life-tables. The consistency of projected GC incidence and all-cause mortality with the population data were confirmed by the Mantel–Cox log-rank test for goodness-of-fit. In simulating the lifetime experience of the target population, Markov models used cohort analysis to calculate the outcomes of expected lifetime cost, lifetime effectiveness and the incremental cost-effectiveness ratio (ICER), which is defined as the additional cost ($) of a specific strategy divided by its additional clinical benefit (QALY) relative to the next least expensive alternative. Based on the ICERs of each strategy, the decision tree suggested the optimal strategy, which is the one with the highest ICER below the Singapore threshold of $46,200/QALY.

One-way deterministic sensitivity analysis was applied to identify the parameters with significant impact on the model. For the clinical and epidemiological parameters, the range for the sensitivity analysis was based on the upper and lower bounds of biological plausibility as reported in the literature. As cost data follow right-skewed distributions [35], base case estimates were halved and doubled to determine the range [6]. We analyzed the net health benefit (NHB) projected by the model to quantify the impact of the input parameters and to identify the associated thresholds for choosing the best strategy. We did not run sensitivity analyses on GC incidence as its variation have been well represented by specific values across age and gender subpopulations.

We conducted probabilistic sensitivity analysis (PSA) to assess the influence of uncertainty surrounding point estimates of input parameters. According to the data informing the point estimates, nine parameters qualified for the PSA, during which 1000 Monte Carlo cycles were exercised on the nine distributions assigned to these parameters (Table 2). The results were summarized in the form of the Cost-Effectiveness Acceptability Frontier which presented the optimal strategy and its associated probability after accounting for the uncertainties jointly contributed by these nine parameters.

**Table 2:** Distributions assigned to parameters in the probabilistic sensitivity analysis.

Input variables	Type of Distribution	Mean (S.D)
Utility Score		
Stage 1	Gamma*	0.88 (0.05)
Stage 2	Gamma*	0.86 (0.07)
Stage 3	Gamma*	0.77 (0.10)
Stage 4	Gamma*	0.68 (0.08)
Odds ratio†	LogNormal*	6.00 (2.46)
Prevalence of premalignant gastric lesions (%)	Beta^1^	13.5 (6.75)
Stage distribution of GC cases(Stage 1:2:3:4)		
Population with OGD follow-up	Dirichlet	85%:4%:8%:3%
Population without OGD follow-up	Dirichlet	7%:17%:33%:43%
Age of starting OGD	Actual distribution	

Methods of moments; S.D: standard deviation.

Odds ratio of GC in high risk group relative to low risk group.

## Results

### Base-case analysis

**Figure 2:**
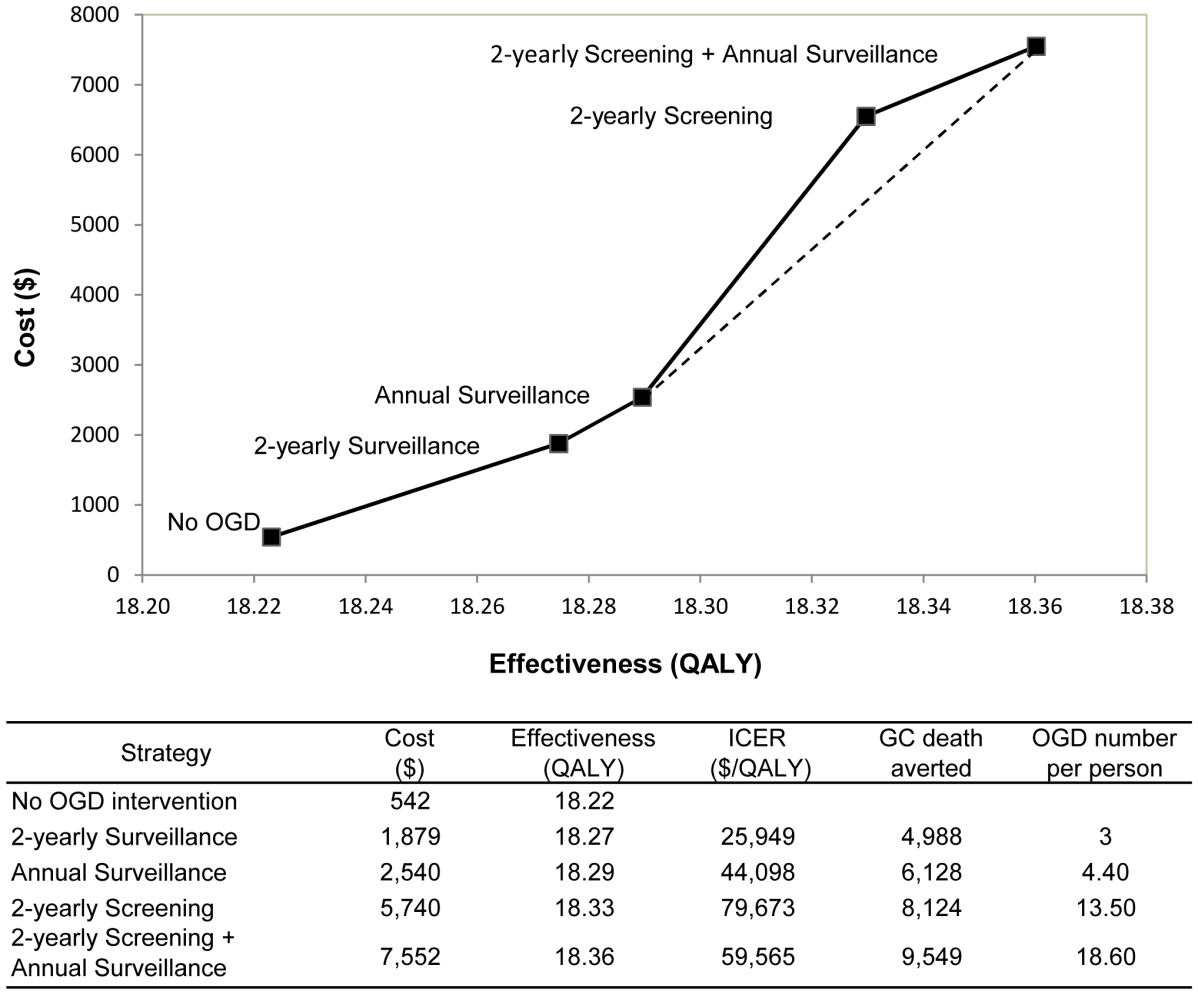
Cost effectiveness analysis of the five strategies at base-case analysis.

Given the Singapore specific WTP of 46,200/QALY, the 2-yearly OGD surveillance and the annual OGD surveillance were both considered cost-effective for the target population. The former was the most cost-effective strategy with the lowest ICER of $25,949/QALY while the latter was the optimal strategy as the annual OGD surveillance was projected to create 0.05 more QALYs and prevent 2,140 more GC deaths than the 2-yearly surveillance strategy (Figure 2). The 2-yearly screening strategy was extendedly dominated by the combination of the annual surveillance strategy and the 2-yearly screening plus annual surveillance strategies.

### Heterogeneity across age and gender subgroups

The performances of the four prevention strategies were different across age-gender subgroups. As in Table 3, each of the age-gender subgroups had its own cost, effectiveness, ICER and the optimal strategy. These variations demonstrated the heterogeneity of the strategies when applied to different risk groups categorized by known factors for GC development. However, the general trend was clear that males featured by higher GC risk [12], [21] were associated with much lower ICERs than female subgroups of the same age. Older age groups generated lower ICERs than those younger age groups independent of their gender. As in base-case analysis, the extended dominance occurred for the 2-yearly screening strategy in all subgroups.

**Table 3:** Heterogeneity of the four strategies by cost, utility and ICER across age and gender subgroups.

			Target	population	(years)		Male	(years)			Female	(years)	
		50–54	55–59	60–64	65–69	50–54	55–59	60–64	65–69	50–54	55–59	60–64	65–69
No OGD Intervention	Cost ($)	475	534	592	632	621	705	784	844	341	380	420	448
	Utility (QALY)	20.49	18.62	16.58	14.43	19.57	17.64	15.54	13.37	21.35	19.53	17.52	15.36
Surveillance	Cost ($)	1892	1879	1854	1804	2002	2015	2000	1968	1795	1764	1725	1664
(2-yearly OGD)	Utility (QALY)	20.54	18.67	16.64	14.5	19.64	17.71	15.63	13.45	21.39	19.57	17.56	15.41
	ICER ($/QALY)	**28,962**	**24,417**	20,856	18,141	21,445	18,809	15,013	12,948	**40,367**	**34,796**	**30,024**	**25,928**
Surveillance	Cost ($)	2640	2554	2451	2321	2716	2655	2561	2449	2579	2477	2361	2219
(annual OGD)	Utility (QALY)	20.55	18.69	16.66	14.51	19.66	17.73	15.65	13.48	21.4	19.58	17.57	15.42
	ICER ($/QALY)	56,328	46,291	**36,598**	**30,753**	**32,290**	25,704	19,958	15,954	94,451	80,694	64,118	56,268
Screening	Cost ($)	6311	5846	5328	4759	6225	5769	5263	4712	6407	5930	5401	4816
(2-yearly OGD)*	Utility (QALY)	20.58	18.72	16.7	14.55	19.71	17.79	15.72	13.56	21.41	19.6	17.59	15.44
	ICER ($/QALY)	114,823	87,827	70,367	60,150	67,000	48,633	40,358	31,338	218,920	177,622	145,464	125,612
Screening	Cost ($)	8191	7671	7081	6413	8114	7621	7036	6406	8268	7744	7146	6485
(2-yearly OGD) +													
Surveillance	Utility (QALY)	20.61	18.76	16.74	14.59	19.75	17.85	15.79	13.64	21.43	19.62	17.63	15.49
(annual OGD)	ICER ($/QALY)	58,829	49,167	37,419	42,124	47,158	**36,578**	**25,541**	**20,470**	129,238	65,964	44,925	33,085

The optimal strategy for each subgroup is highlighted in bold font.

### Sensitivity analysis

Our model was found to be sensitive to eight parameters that were each able to cause a minimum of 0.2 QALY change within their clinical ranges. The relationship between these influential parameters and the NHBs predicted for each strategy is summarized in Table 4. As anticipated *a priori*, the discount rate, age of starting surveillance, cost of follow-up OGD and proportion of program cost were negatively correlated to the NHBs. The odds ratio for GC of the high risk group, prevalence of premalignant lesions, utility of GC Stage 1 and early detection effect in the interval years of the 2-yearly surveillance program had positive relationships with the model NHBs.

**Table 4:** Influential parameters and their thresholds for the choice of optimal strategy.

Input parameters	Range	Relationship with	Thresholds and	the corresponding	optimal strategy
		model NHBs			
Discount rate (%)	3–5	negative	3–3.20		3.20–5
			annual surveillance		2-yearly surveillance
Age (year)	50–69	negative	50–57	57–64	64–69
			2-yearly surveillance	annual surveillance	screening + surveillance
Program cost Proportion (%)	20–80	negative	20–43		43–80
			annual surveillance		2-yearly surveillance
Cost of follow-up OGD ($)	170–680	negative	170–208,	208–356	356–680
			surveillance + screening	annual surveillance	2-yearly surveillance
Utility of GC Stage 1	0.6–1	positive	0.6–0.85		0.85–1
			2-yearly surveillance		annual surveillance
Odds ratio of high risk subjects	2.4–21.5	positive	2.4–5.46		5.46–21.5
			2-yearly surveillance		annual surveillance
Prevalence of premalignancy (%)	6.8–40	positive	6.8–14.97		14.97–40
			annual surveillance		2-yearly surveillance
Early detection for surveillance	40–90	positive	40–62		62–90
program during interval years (%)			annual surveillance		2-yearly surveillance

These influential parameters also had a strong impact on the choice of optimal strategy. They were identified with one or two cut-off values defining specific ranges where the optimal strategy differed (Table 4). The matrix of influential parameters and their thresholds has great implications in designing and operating an actual healthcare program. For example, for odds ratio which reflects the GC risk associated with precancerous lesions of high risk subjects [23], our model identified a threshold of 5.46. This finding implies that a subpopulation with a GC risk of 5.46 times that of a healthy person should undergo annual OGD surveillance, whereas a subpopulation with a GC risk of between 2.4 and 5.46 favored alternate OGD surveillance. Follow-up OGD was the essential clinical service offered by the preventive strategies. Offering this service at a cost below $208 would make the most intensive strategy, 2-yearly screening plus annual surveillance, the optimal strategy. If this cost exceeds $356, the least intensive strategy of 2-yearly surveillance would be the optimal strategy.

**Figure 3:**
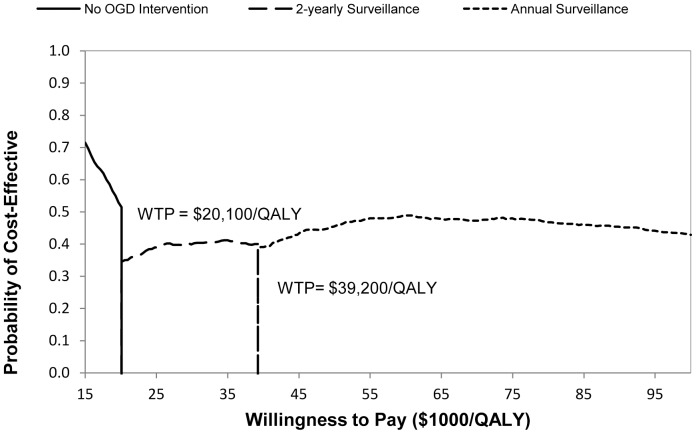
Cost Effectiveness Acceptability Frontier of the optimal strategies.

PSA in our study helped to identify the optimal strategy and its associated probability given the officially defined WTPs. As shown in the Cost Effectiveness Acceptability Frontier (Figure 3), the choice of optimal strategy evolved with increasing WTP. At the Singapore WTP of $46,200/QALY, the expected optimal strategy was the annual surveillance, which was consistent with the base-case analysis. However, the finding was not definite but with a probability of 44.5% after accounting for the model uncertainty. Below the threshold of $20,100/QALY as reported in previous Asian studies [5], [6], none of the evaluated strategies was preferred over no OGD intervention. The 2-yearly OGD surveillance strategy started to demonstrate its advantage over others between $20,100/QALY and $39,200/QALY. For the most commonly used WTP of $50,000/QALY in advanced countries [40], [41], annual surveillance remained the optimal strategy.

## Discussion

To the best of our knowledge, a state-funded GC surveillance program has never been officially established in any country of the world. Singapore, with its advanced health care system and a small population, is an ideal place for implementation of such a program and may well be used as a model for other jurisdictions, such as countries with low to intermediate GC risk. In recent years, Singapore has systematically explored the feasibility of an OGD-based surveillance program for GC. A series of studies have been launched to address the practical issues such as the cost efficiency of delivering GC surveillance [31], quality of life in GC patients [19] and the long-term outcome of high risk subjects [15]. Synthesizing these recent findings, our study contributed cost-effectiveness data to the area of GC surveillance. These studies taken together are very helpful for the implementation of an evidence-based surveillance program for GC in Singapore.

As suggested by the Asia-Pacific consensus guidelines, it is not feasible to screen the general population in a country with low to intermediate GC incidence and mortality [42]. For these countries, surveillance focusing on high risk subjects has emerged as a promising alternative. Excluding the majority of low risk subjects who may not develop GC during their lifetime, surveillance intuitively represents a strategy of resource-saving with little compromise in health gain. Our model evaluated four prevention strategies, in order of increasing resource utilization: the 2-yearly surveillance, the annual surveillance, the 2-yearly screening and the 2-yearly screening plus annual surveillance. The two surveillance programs were cost-effective for our target population of Singaporean Chinese aged 50-69 years old with an intermediate GC risk, whereas the strategies based on universal screening were either extendedly dominated or cost-ineffective. Changing from annual surveillance to 2-yearly screening produced the biggest incremental cost. Moreover, the lifetime number of OGD examinations tripled from 4.4 to 13.5 per subject (Figure 2). Therefore, population-based screening would most certainly cause a strain on many health care systems, for example, the insufficient supply of facilities and qualified endoscopists as occurred in Japan [43]. Focused surveillance, however, tends to be structured as a hospital-based service, which has shown to be practical and efficient due to easy subject recruitment and participation [44]. Delivering endoscopic GC surveillance through a hospital-based structure has been proven effective and cost-effective in multiple populations [7], [10].

Annual OGD surveillance overall was found to be the optimal strategy. However, it may not be the one-for-all solution given the uneven distribution of GC risk in the target population. Gastric cancer incidence is known to be related to age and gender [12], [21]. Therefore the heterogeneity of the four strategies in response to age and gender is expected and consequently the choice of optimal strategy is different. This heterogeneity is closely relevant to resource allocation and priority setting from a perspective of program implementation. In line with the economic principle that lower ICERs indicate better return on investment, resources should be prioritized to male subgroups and older subjects. In particular, the 65-69 year old males with the lowest ICER should be favorably considered (Table 3). This finding is consistent with a previous model suggesting that the age of 65 years is the optimal age to start OGD follow-up [13].

In a country with high GC risk, it appears an easy decision that population-based screening is the best strategy in prevention of GC. This issue becomes more complicated for countries with a relatively lower GC burden. As our target population is at an intermediate GC risk, we evaluated the four strategies including both screening and surveillance and conducted a comprehensive sensitivity analysis. The matrix of influential parameters and their respective thresholds (Table 4) illustrated the conditions or requirements for the individual strategies to be cost-effective within the Singapore context. With such an informative matrix, choosing the optimal strategy for a given health care system becomes a matter of modifying influential factors and achieving certain thresholds. In our study the cost of follow-up OGD was found to be influential, which was consistent with the models by Dan et al [13] and Gupta et al [45]. Its price of $340 was negotiated between the GCEP and the National University Hospital and was cheaper than the normal hospital rate [31]. Another influential factor is the proportion of program cost indicating the operational efficiency of an actual program [33]. Our model projected that annual surveillance is the preferred strategy conditional on an efficient control of program cost below 43% of the total operating budget. Otherwise, the 2-yearly surveillance has to be chosen, which would then produce less health life years than the annual surveillance strategy.

It remains controversial what gastric lesions are amenable to continuing GC surveillance. Following Correa’s model of GC genesis, atrophic gastritis, intestinal metaplasia and dysplasia are commonly perceived as precancerous lesions and therefore have been suggested for OGD follow-up [16]. However, limiting the target gastric premalignancies to these intermediate steps of the Correa’s model may not fully realize the preventive potential of OGD surveillance. In fact, other subgroups have also been suggested for OGD follow-up, for example, gastric ulcer [9], first-line relatives of the GC patient [46], people with blood pepsinogen levels below 3mg/ml [47] or with certain genetic polymorphisms [48]. The issue underlying the controversy is what degree of GC risk justifies continuing OGD follow-up. In our study, we believe that any traits which predispose certain subgroups to additional GC risk deserve further investigation. Therefore, our model used odds ratio with a wide range of 2.4 to 21.5 to represent excessive GC risk attributable to various predisposing factors. The sensitivity analysis identified a threshold of 5.46, below which the 2-yearly surveillance should be recommended, or the annual surveillance is the optimal strategy in Singapore. This finding does not address the issue of the appropriate lesions for OGD surveillance. It reflects a fundamental principle of economic evaluation, which is to recommend the right strategy to the right population based on cost-effective ratios.

When building our models for the surveillance strategies, we did not use progression rates from other studies. Instead, we used the epidemiological profile of the target population and the odds ratio of high risk subjects to generate progression rates of GC for low and high risk subgroups. There are good reasons to do so. The epidemiological profile refers to two components; the GC incidence which is positively associated with the cost-effectiveness of a preventive strategy, and background mortality which exerts a negative influence due to competing diseases [49]. Incorporating the epidemiological profile this way makes our models not only Singapore-relevant, but also adaptable to other jurisdictions by simply inputting the epidemiological profile of the local population. Another reason is that most progression rates are estimated in populations at high GC risk [50], [51]. However, our model is framed for a population with low to intermediate GC risk. Therefore, transferring these rates into our model will over-estimate the GC risk. A systematic review on economic evaluations of endoscopic surveillance of precancerous lesions concluded that conflicting results from these studies were caused by heterogeneity in the progression rates assumed in their models [52].

Making a decision solely based on the expected cost-effectiveness ratios is premature as the likelihood associated with these ratios is also valuable for an informed decision [53]. At the Singapore WTP of $46,200/QALY, the result that annual surveillance is the optimal strategy is strengthened by the same recommendation from the PSA. Additionally PSA estimated a probability of 44.5% illustrating how confident we are in the above decision after accounting for the model uncertainties. The uncertainty is an inevitable element in decision-making.

A few strengths about this study are noted. Both utility and cost data were obtained from our studies on the target population thereby improving the internal validity of our model. Unlike other economic evaluations [8]–[10], we used the epidemiological profile and odds ratio to estimate the progression rate of high risk subjects for the surveillance strategies, which has increased the generalizability of our model.

Nevertheless, the study does have some limitations. To mitigate the lead-time bias and length-time bias in the models simulating the preventive strategies, we assumed the same survival experience of GC patients for all the five strategies. The Markov structures were also adjusted to ensure consistent GC incidences in each model. As a result, extra survival time due to these two types of bias was alleviated. However we cannot completely rule out their existence. The compliance rate with the OGD schedule was assumed to be 100% which is unlikely in reality [54], [55]. However, it is less likely that the model validity would be affected. The current study aims to provide a conceptual assessment of the cost-effectiveness potential of the surveillance and screening strategies for future program implementation. To this end, our model has provided useful data to avoid conceptual deficit [56].

## Conclusion

Endoscopic surveillance has the potential to be a cost-effective strategy in prevention of GC for populations with low to intermediate risk. It is necessary for policy-makers to evaluate multiple strategies for the purpose of recommending the appropriate strategy to certain subgroups. In implementing an endoscopic surveillance program, influential factors have to be identified and evaluated to achieve cost-effectiveness in a given health care system and target population.

## Supporting Information

Figure S1 **Markov model of the no intervention strategy.**
(TIF)

Figure S2 **Markov model of the annual surveillance strategy.**
(TIF)

Figure S3 **Markov model of the 2-yearly surveillance strategy.**
(TIF)

Figure S4 **Markov model of the 2-yearly screening strategy.**
(TIF)

Figure S5 **Markov model of the 2-yearly surveillance plus annual surveillance strategy.**
(TIF)

Figure S6 **Decision tree comparing the five Markov models.**
(TIF)

Table S1 **Algorithm of stage-specific gastric cancer treatment.**
(DOCX)

## References

[1] Lee KJ, Inoue M, Otani T, Iwasaki M, Sasazuki S, et al (2006) Gastric cancer screening and subsequent risk of gastric cancer: a large-scale population-based cohort study, with a 13-year follow-up in Japan. Int J Cancer 118: 2315–2321. 16331632 10.1002/ijc.21664

[2] Miyamoto A, Kuriyama S, Nishino Y, Tsubono Y, Nakaya N, et al (2007) Lower risk of death from gastric cancer among participants of gastric cancer screening in Japan: a population-based cohort study. Prev Med 44: 12–19. 16956654 10.1016/j.ypmed.2006.07.016

[3] Hosokawa O, Miyanaga T, Kaizaki Y, Hattori M, Dohden K, et al (2008) Decreased death from gastric cancer by endoscopic screening: association with a population-based cancer registry. Scand J Gastroenterol 43: 1112–1115. 18609154 10.1080/00365520802085395

[4] Tsuji I, Tsubono Y, Hisamichi S (2001) Effectiveness and cost-benefit of screening for gastric cancer in Japan. Nihon Rinsho 59 Suppl 4533–537. 11424440

[5] Lee YC, Lin JT, Wu HM, Liu TY, Yen MF, et al (2007) Cost-effectiveness analysis between primary and secondary preventive strategies for gastric cancer. Cancer Epidemiol Biomarkers Prev 16: 875–885. 17507609 10.1158/1055-9965.EPI-06-0758

[6] Chang HS, Park EC, Chung W, Nam CM, Choi KS, et al (2012) Comparing endoscopy and upper gastrointestinal X-ray for gastric cancer screening in South Korea: a cost-utility analysis. Asian Pac J Cancer Prev 13: 2721–2728. 22938448 10.7314/apjcp.2012.13.6.2721

[7] Whiting JL, Sigurdsson A, Rowlands DC, Hallissey MT, Fielding JW (2002) The long term results of endoscopic surveillance of premalignant gastric lesions. Gut 50: 378–381. 11839718 10.1136/gut.50.3.378 PMC1773155

[8] Yeh JM, Hur C, Kuntz KM, Ezzati M, Goldie SJ (2010) Cost-effectiveness of treatment and endoscopic surveillance of precancerous lesions to prevent gastric cancer. Cancer 116: 2941–2953. 20564399 10.1002/cncr.25030 PMC2946062

[9] Yeh JM, Ho W, Hur C (2010) Cost-effectiveness of endoscopic surveillance of gastric ulcers to improve survival. Gastrointest Endosc 72: 33–43. 20430384 10.1016/j.gie.2010.01.047 PMC2902548

[10] Dinis-Ribeiro M, da Costa-Pereira A, Lopes C, Moreira-Dias L (2007) Feasibility and cost-effectiveness of using magnification chromoendoscopy and pepsinogen serum levels for the follow-up of patients with atrophic chronic gastritis and intestinal metaplasia. J Gastroenterol Hepatol 22: 1594–1604. 17845687 10.1111/j.1440-1746.2007.04863.x

[11] Hassan C, Zullo A, Di Giulio E, Annibale B, Lahner E, et al (2010) Cost-effectiveness of endoscopic surveillance for gastric intestinal metaplasia. Helicobacter 15: 221–226. 20557364 10.1111/j.1523-5378.2010.00752.x

[12] Singapore Cancer Registry Committee (2012) Trends in cancer incidence in Singapore 1968–2007. singapore. 191 p.

[13] Dan YY, So JB, Yeoh KG (2006) Endoscopic screening for gastric cancer. Clin Gastroenterol Hepatol 4: 709–716. 16765306 10.1016/j.cgh.2006.03.025

[14] Xie F, Luo N, Blackhouse G, Goeree R, Lee HP (2008) Cost-effectiveness analysis of Helicobacter pylori screening in prevention of gastric cancer in Chinese. Int J Technol Assess Health Care 24: 87–95. 18218173 10.1017/S0266462307080117

[15] Zhu F, Loh M, Hill J, Lee S, Koh KX, et al (2009) Genetic factors associated with intestinal metaplasia in a high risk Singapore-Chinese population: a cohort study. BMC Gastroenterol 9: 76. 19822020 10.1186/1471-230X-9-76 PMC2766386

[16] Dinis-Ribeiro M, Areia M, de Vries AC, Marcos-Pinto R, Monteiro-Soares M, et al (2012) Management of precancerous conditions and lesions in the stomach (MAPS): guideline from the European Society of Gastrointestinal Endoscopy (ESGE), European Helicobacter Study Group (EHSG), European Society of Pathology (ESP), and the Sociedade Portuguesa de Endoscopia Digestiva (SPED). Endoscopy 44: 74–94. 22198778 10.1055/s-0031-1291491 PMC3367502

[17] Tsubono Y, Nishino Y, Tsuji I, Hisamichi S (2000) Screening for Gastric Cancer in Miyagi, Japan: Evaluation with a Population-Based Cancer Registry. Asian Pac J Cancer Prev 1: 57–60. 12718689

[18] Wong BC, Lam SK, Wong WM, Chen JS, Zheng TT, et al (2004) Helicobacter pylori eradication to prevent gastric cancer in a high-risk region of China: a randomized controlled trial. JAMA 291: 187–194. 14722144 10.1001/jama.291.2.187

[19] Zhou HJ, So JB, Yong WP, Luo N, Zhu F, et al (2012) Validation of the functional assessment of cancer therapy-gastric module for the Chinese population. Health Qual Life Outcomes 10: 145. 23194009 10.1186/1477-7525-10-145 PMC3520860

[20] Weinstein MC, Siegel JE, Gold MR, Kamlet MS, Russell LB (1996) Recommendations of the Panel on Cost-effectiveness in Health and Medicine. JAMA : the journal of the American Medical Association 276: 1253–1258. 8849754

[21] Department of Statistics Singapore (2012) Yearbook of Statistics Singapore 2012. Singapore Ministry of Trade & Industry, Republic of Singapore. 319 p.

[22] U.S. Environmental Protection Agency (2010) Cost of Illness Hand book 1st ed. Pennsylvania US Environmental Protection Agency.

[23] Watabe H, Mitsushima T, Yamaji Y, Okamoto M, Wada R, et al (2005) Predicting the development of gastric cancer from combining Helicobacter pylori antibodies and serum pepsinogen status: a prospective endoscopic cohort study. Gut 54: 764–768. 15888780 10.1136/gut.2004.055400 PMC1774550

[24] Ang TL, Fock KM, Dhamodaran S, Teo EK, Tan J (2005) Racial differences in Helicobacter pylori, serum pepsinogen and gastric cancer incidence in an urban Asian population. J Gastroenterol Hepatol 20: 1603–1609. 16174081 10.1111/j.1440-1746.2005.03898.x

[25] Kim YS, Park HA, Kim BS, Yook JH, Lee MS (2000) Efficacy of screening for gastric cancer in a Korean adult population: a case-control study. J Korean Med Sci 15: 510–515. 11068986 10.3346/jkms.2000.15.5.510 PMC3054678

[26] Kubota H, Kotoh T, Masunaga R, Dhar DK, Shibakita M, et al (2000) Impact of screening survey of gastric cancer on clinicopathological features and survival: retrospective study at a single institution. Surgery 128: 41–47. 10876184 10.1067/msy.2000.106812

[27] Nakashima H, Nagahama R, Yamamoto T, Ohkura Y (2010) Mass screening for gastric cancer: how to select patients for endoscopic examination. Gastric Cancer 13: 78–83. 20602193 10.1007/s10120-009-0538-3

[28] Voutilainen ME, Juhola MT (2005) Evaluation of the diagnostic accuracy of gastroscopy to detect gastric tumours: clinicopathological features and prognosis of patients with gastric cancer missed on endoscopy. Eur J Gastroenterol Hepatol 17: 1345–1349. 16292088 10.1097/00042737-200512000-00013

[29] Environmental Protection Agency USA (2000) Cost of Stomach Cancer. Cost of Illness Handbook. washington DC: Environmental Protection Agency USA. pp. 1–45.

[30] Morabito A, Carillio G, Longo R (2009) Systemic treatment of gastric cancer. Crit Rev Oncol Hematol 70: 216–234. 18829344 10.1016/j.critrevonc.2008.08.005

[31] Zhou HJ, Li SC, Naidoo N, Zhu F, Yeoh KG (2013) Empirical evidence of the continuing improvement in cost efficiency of an endoscopic surveillance programme for gastric cancer in Singapore from 2004 to 2010. BMC Health Serv Res 13: 139. 23587354 10.1186/1472-6963-13-139 PMC3637081

[32] Centers for Disease Control and Prevention NBCCEDP (2005) Policies and Procedures manual. Atlanta: Centers Disease Control & Prevention,.

[33] Subramanian S, Tangka FKL, Hoover S, DeGroff A, Royalty J, et al (2011) Clinical and programmatic costs of implementing colorectal cancer screening: Evaluation of five programs. Evaluation and program planning 34: 147–153. 21036399 10.1016/j.evalprogplan.2010.09.005

[34] Commission on Macroeconomics and Health (2001) Macroeconomics and Health: Investing in Health for Economic Development. Geneva: World Health Organization.

[35] Thompson SG, Barber JA (2000) How should cost data in pragmatic randomised trials be analysed? BMJ 320: 1197–1200. 10784550 10.1136/bmj.320.7243.1197 PMC1127588

[36] Koong HN, Chan HS, Nambiar R, Soo KC, Ho J, et al (1996) Gastric cancers in Singapore: poor prognosis arising from late presentation. Aust N Z J Surg 66: 813–815. 8996060 10.1111/j.1445-2197.1996.tb00755.x

[37] Wai CT, Yeoh KG, Ho KY, Kang JY, Lim SG (2002) Diagnostic yield of upper endoscopy in Asian patients presenting with dyspepsia. Gastrointest Endosc 56: 548–551. 12297772 10.1067/mge.2002.128493

[38] Robert G, Brown J, Garvican L (2000) Cost of quality management and information provision for screening: colorectal cancer screening. Journal of medical screening 7: 31–34. 10807144 10.1136/jms.7.1.31

[39] Weinstein MC, O'Brien B, Hornberger J, Jackson J, Johannesson M, et al (2003) Principles of good practice for decision analytic modeling in health-care evaluation: report of the ISPOR Task Force on Good Research Practices—Modeling Studies. Value Health 6: 9–17. 12535234 10.1046/j.1524-4733.2003.00234.x

[40] Xie F, O'Reilly D, Ferrusi IL, Blackhouse G, Bowen JM, et al (2009) Illustrating economic evaluation of diagnostic technologies: comparing Helicobacter pylori screening strategies in prevention of gastric cancer in Canada. J Am Coll Radiol 6: 317–323. 19394572 10.1016/j.jacr.2009.01.022

[41] Shiroiwa T, Sung YK, Fukuda T, Lang HC, Bae SC, et al (2010) International survey on willingness-to-pay (WTP) for one additional QALY gained: what is the threshold of cost effectiveness? Health Econ 19: 422–437. 19382128 10.1002/hec.1481

[42] Fock KM, Talley N, Moayyedi P, Hunt R, Azuma T, et al (2008) Asia-Pacific consensus guidelines on gastric cancer prevention. J Gastroenterol Hepatol 23: 351–365. 18318820 10.1111/j.1440-1746.2008.05314.x

[43] Leung WK, Wu MS, Kakugawa Y, Kim JJ, Yeoh KG, et al (2008) Screening for gastric cancer in Asia: current evidence and practice. Lancet Oncol 9: 279–287. 18308253 10.1016/S1470-2045(08)70072-X

[44] Chien PF, Khan KS (2001) Evaluation of a clinical test. II: Assessment of validity. BJOG 108: 568–572. 11426889 10.1111/j.1471-0528.2001.00128.x

[45] Gupta N, Bansal A, Wani SB, Gaddam S, Rastogi A, et al.. (2011) Endoscopy for upper GI cancer screening in the general population: a cost-utility analysis. Gastrointest Endosc 74: 610–624 e612. 10.1016/j.gie.2011.05.001 21741639

[46] Kang JM, Shin DW, Kwon YM, Park SM, Park MS, et al (2011) Stomach cancer screening and preventive behaviors in relatives of gastric cancer patients. World J Gastroenterol 17: 3518–3525. 21941419 10.3748/wjg.v17.i30.3518 PMC3163250

[47] Mukoubayashi C, Yanaoka K, Ohata H, Arii K, Tamai H, et al (2007) Serum pepsinogen and gastric cancer screening. Intern Med 46: 261–266. 17379991 10.2169/internalmedicine.46.6181

[48] El-Omar EM, Carrington M, Chow WH, McColl KE, Bream JH, et al (2000) Interleukin-1 polymorphisms associated with increased risk of gastric cancer. Nature 404: 398–402. 10746728 10.1038/35006081

[49] Walter LC, Lindquist K, Nugent S, Schult T, Lee SJ, et al (2009) Impact of age and comorbidity on colorectal cancer screening among older veterans. Ann Intern Med 150: 465–473. 19349631 10.7326/0003-4819-150-7-200904070-00006 PMC3769097

[50] Liu CY, Wu CY, Lin JT, Lee YC, Yen AM, et al (2006) Multistate and multifactorial progression of gastric cancer: results from community-based mass screening for gastric cancer. J Med Screen 13 Suppl 1S2–5. 17227633

[51] Ohata H, Kitauchi S, Yoshimura N, Mugitani K, Iwane M, et al (2004) Progression of chronic atrophic gastritis associated with Helicobacter pylori infection increases risk of gastric cancer. Int J Cancer 109: 138–143. 14735480 10.1002/ijc.11680

[52] Areia M, Carvalho R, Cadime AT, Rocha Goncalves F, Dinis-Ribeiro M (2013) Screening for gastric cancer and surveillance of premalignant lesions: a systematic review of cost-effectiveness studies. Helicobacter 18: 325–337. 23566268 10.1111/hel.12050

[53] Barton GR, Briggs AH, Fenwick EA (2008) Optimal cost-effectiveness decisions: the role of the cost-effectiveness acceptability curve (CEAC), the cost-effectiveness acceptability frontier (CEAF), and the expected value of perfection information (EVPI). Value Health 11: 886–897. 18489513 10.1111/j.1524-4733.2008.00358.x

[54] Kwon YM, Lim HT, Lee K, Cho BL, Park MS, et al (2009) Factors associated with use of gastric cancer screening services in Korea. World J Gastroenterol 15: 3653–3659. 19653344 10.3748/wjg.15.3653 PMC2721240

[55] Choi KS, Kwak MS, Lee HY, Jun JK, Hahm MI, et al (2009) Screening for gastric cancer in Korea: population-based preferences for endoscopy versus upper gastrointestinal series. Cancer Epidemiol Biomarkers Prev 18: 1390–1398. 19383892 10.1158/1055-9965.EPI-08-0940

[56] Shapiro J (1982) Evaluation as theory testing: An example from Head Start. Educational Evaluation and Policy Analysis,: 341–353.

