# Supplementary figures and images for: A Cost-Effectiveness Analysis Evaluating Endoscopic Surveillance for Gastric Cancer for Populations with Low to Intermediate Risk
Source: PLoS One. 2013 Dec 27;8(12):e83959. doi: 10.1371/journal.pone.0083959 (PMC3873968; doi:10.1371/journal.pone.0083959)

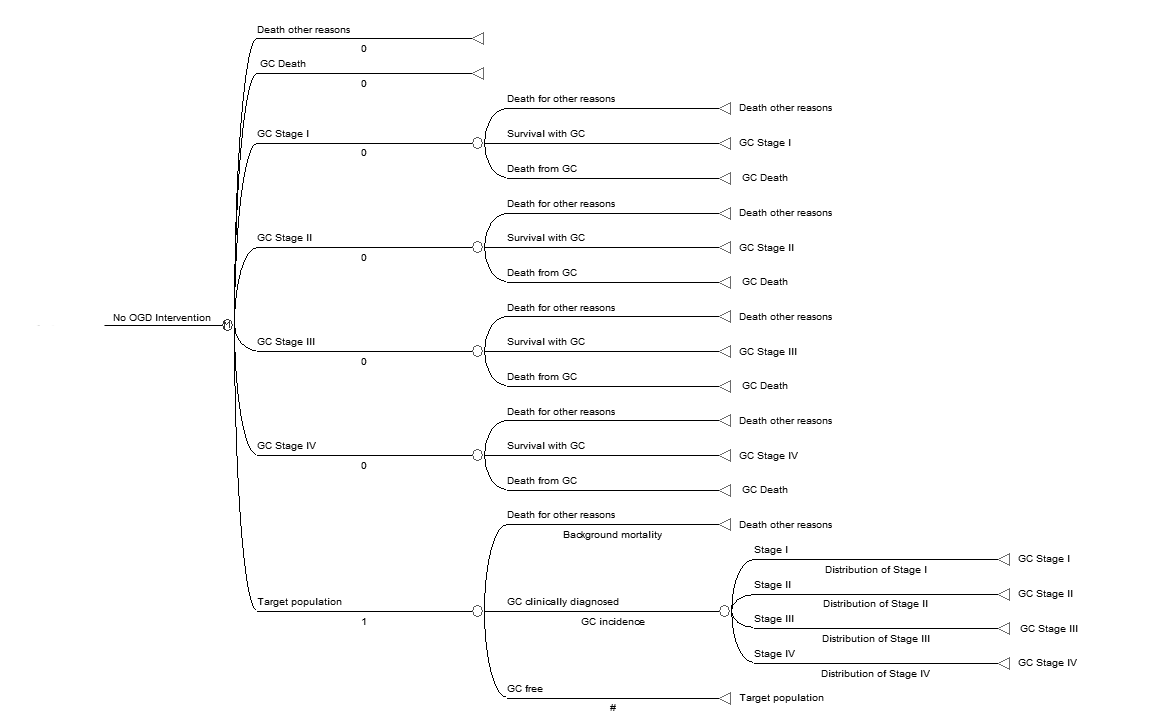

Supplement: Figure S1 — Markov model of the no intervention strategy. (TIF) [file pone.0083959.s001.tif]

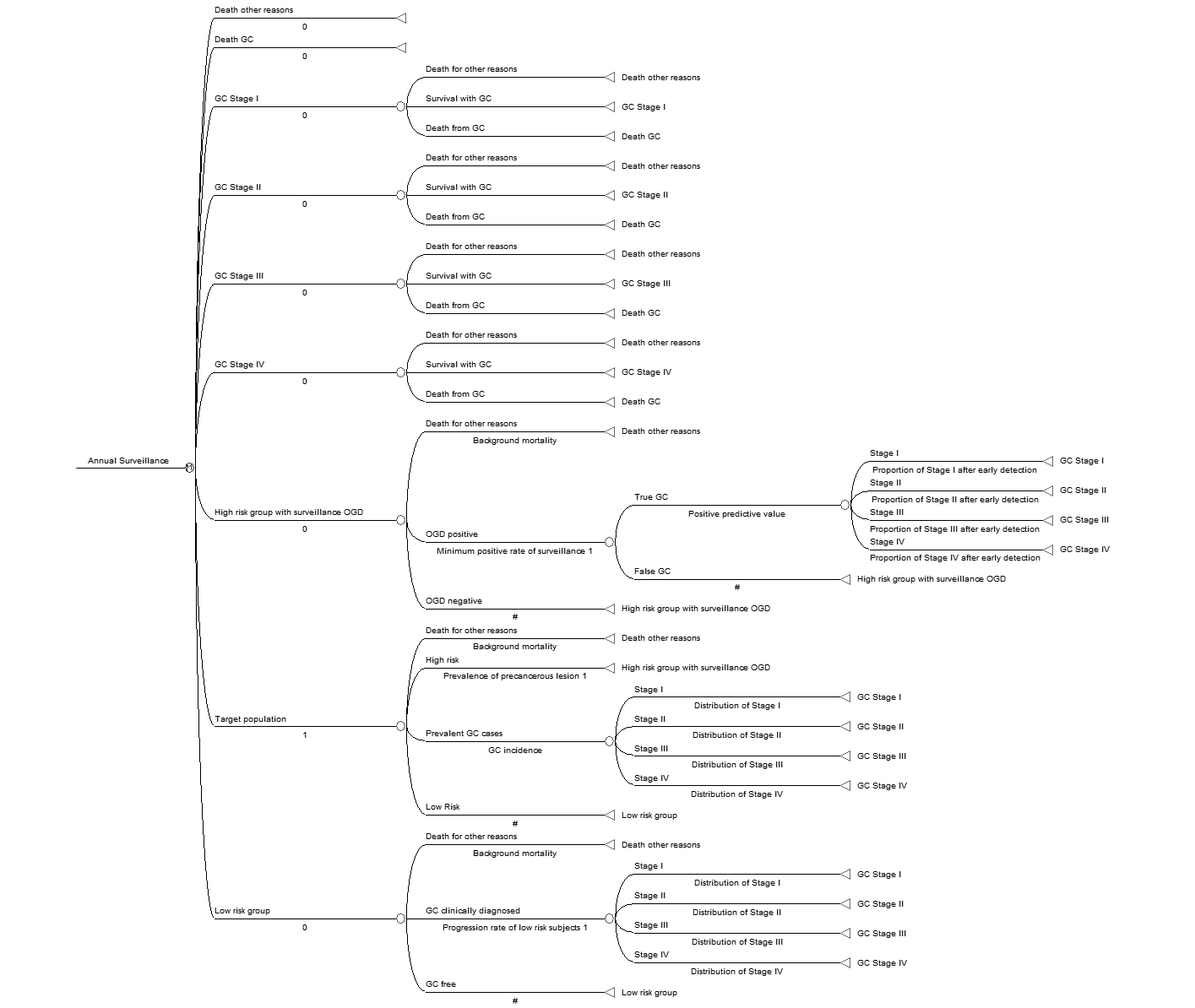

Supplement: Figure S2 — Markov model of the annual surveillance strategy. (TIF) [file pone.0083959.s002.tif]

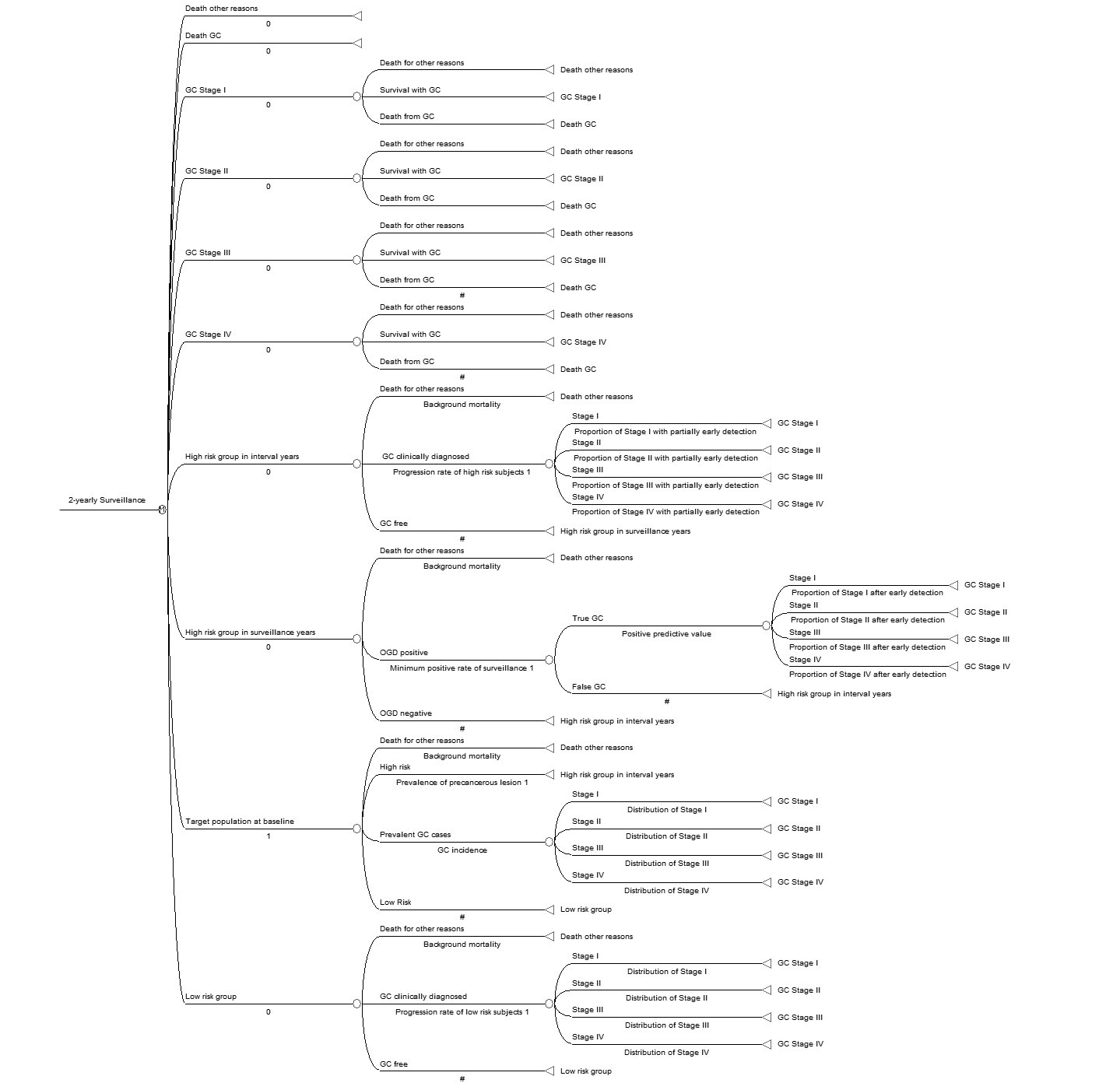

Supplement: Figure S3 — Markov model of the 2-yearly surveillance strategy. (TIF) [file pone.0083959.s003.tif]

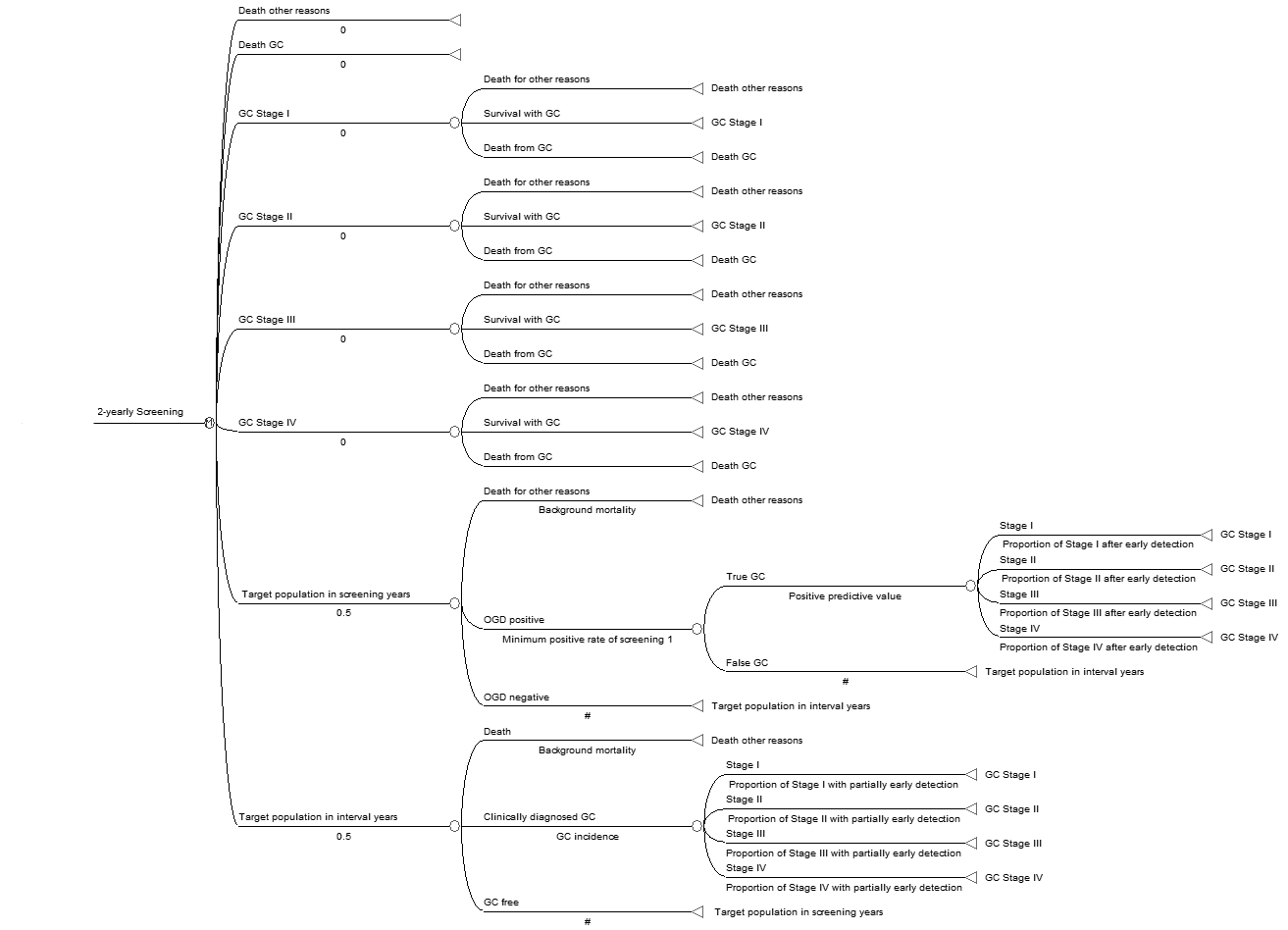

Supplement: Figure S4 — Markov model of the 2-yearly screening strategy. (TIF) [file pone.0083959.s004.tif]

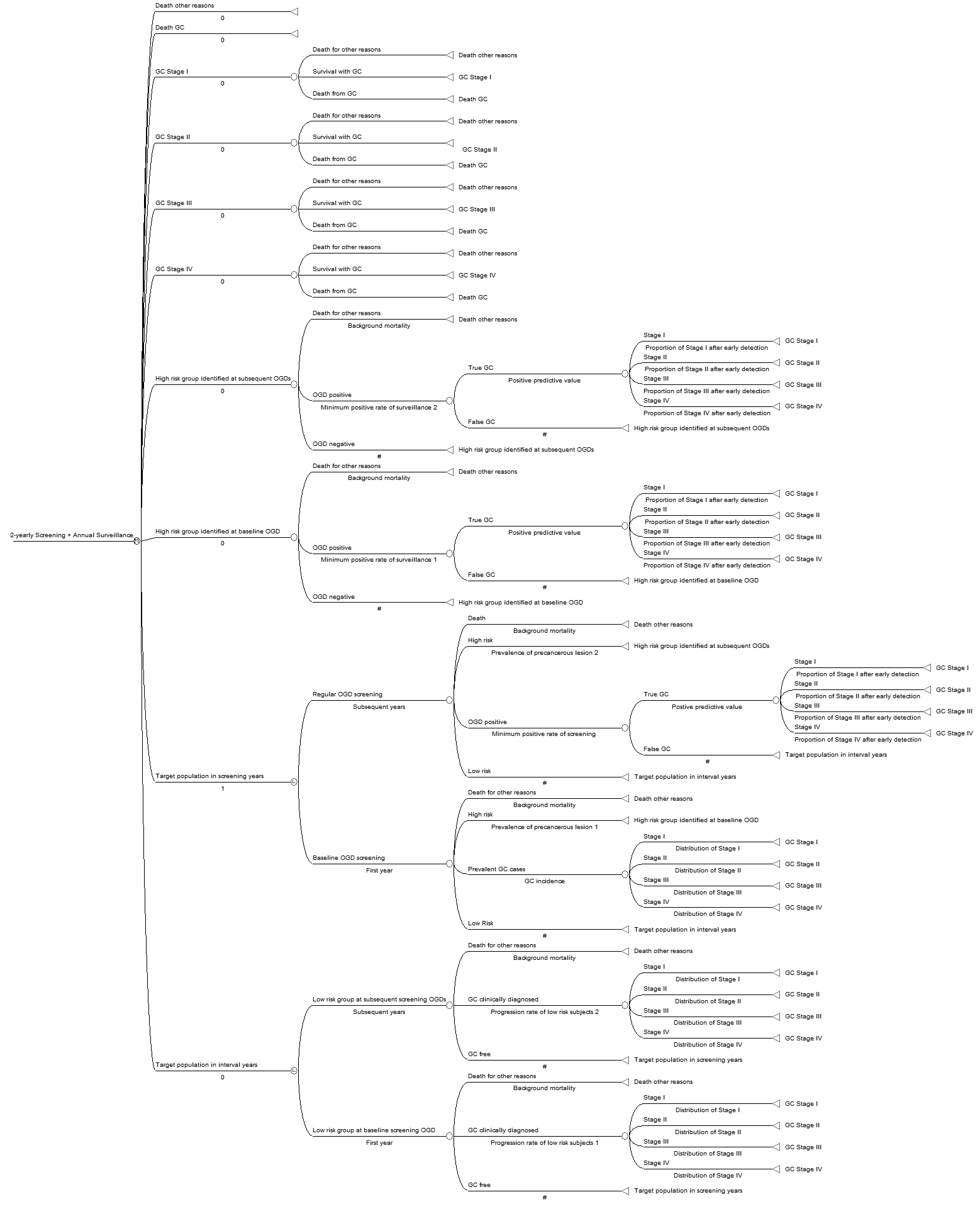

Supplement: Figure S5 — Markov model of the 2-yearly surveillance plus annual surveillance strategy. (TIF) [file pone.0083959.s005.tif]

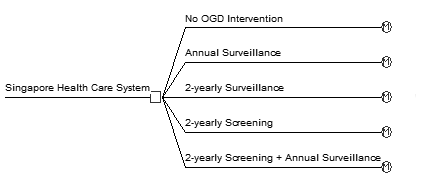

Supplement: Figure S6 — Decision tree comparing the five Markov models. (TIF) [file pone.0083959.s006.tif]
