# Supplementary material for: A Cost-Effectiveness Analysis Evaluating Endoscopic Surveillance for Gastric Cancer for Populations with Low to Intermediate Risk
Source: PLoS One. 2013 Dec 27;8(12):e83959. doi: 10.1371/journal.pone.0083959 (PMC3873968; doi:10.1371/journal.pone.0083959)
Supplement: Table S1 — Algorithm of stage-specific gastric cancer treatment. (DOCX) [file pone.0083959.s007.docx]

**Table 1.** Algorithm of stage specific gastric cancer treatment

| Stage | Medical Components | Medical Service Mix |
| --- | --- | --- |
| Stage 1 | Endoscopic mucosal resection/ Endoscopic submucosa dissection | 30% |
|  | Total/subtotal gastrectomy | 70% |
| Stage 2a | Total/subtotal gastrectomy | 60% |
| Stage 2b | Total/subtotal gastrectomy + chemotherapy | 40% |
| Stage 3 | Total/subtotal gastrectomy + chemotherapy | 100% |
| Stage 4 | Basic support care | 30% |
|  | Bypass surgery + chemotherapy | 30% |
|  | Chemotherapy | 40% |
